# Supplementary material for: Assessment of efficacy of mutagenesis of gamma-irradiation in plant height and days to maturity through expression analysis in rice
Source: PLoS One. 2021 Jan 15;16(1):e0245603. doi: 10.1371/journal.pone.0245603 (PMC7810314; doi:10.1371/journal.pone.0245603)
Supplement: S1 Fig — (PDF) [file pone.0245603.s001.pdf]

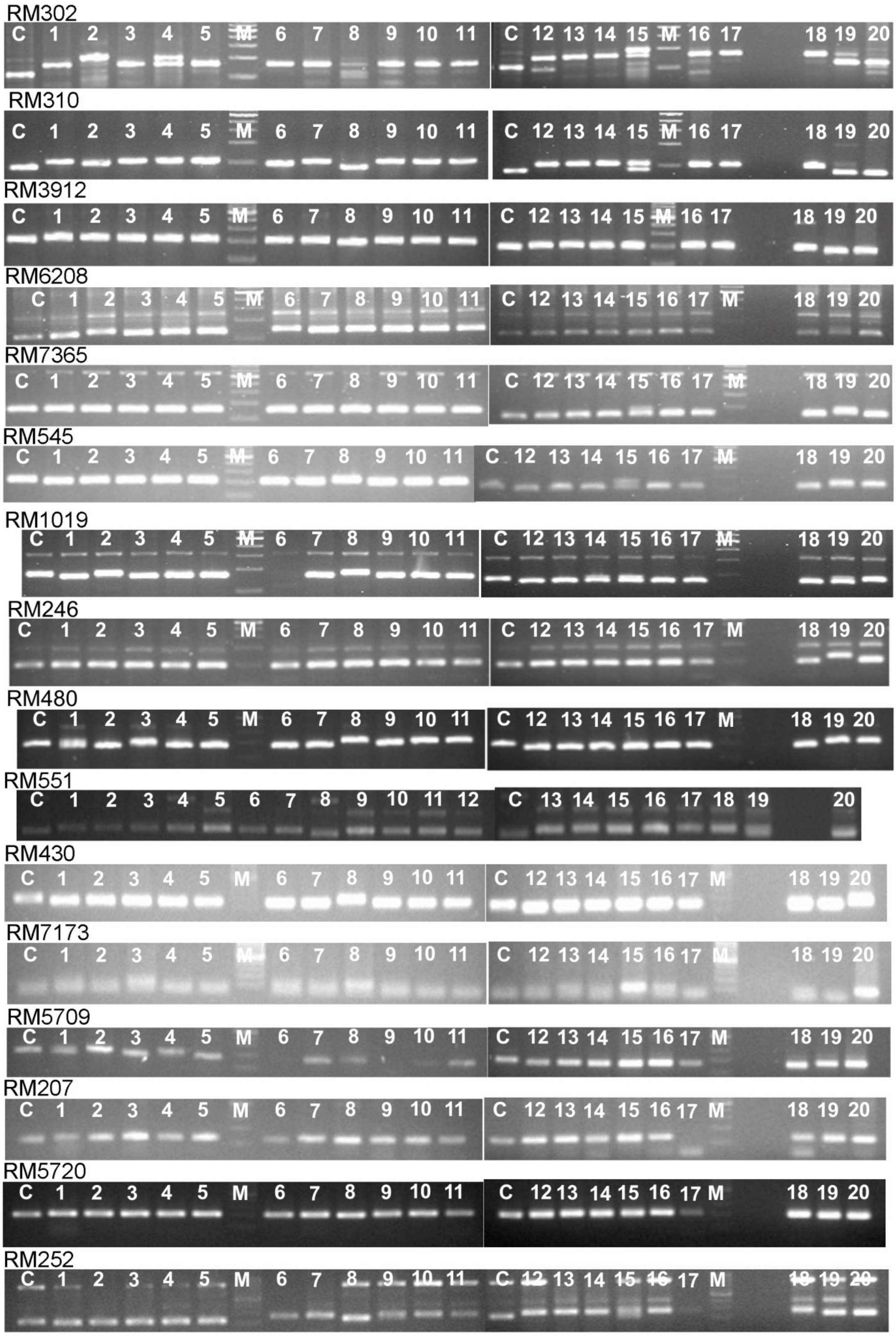
RM302
RM310
RM3912
RM6208
RM7365
RM545
RM1019
RM246
RM480
RM551
RM430
RM7173
RM5709
RM207
RM5720
RM252

31

S1 Fig. Electrophoresis images of polymorphic SSR markers used in the study

Electrophoresis images and amplicons of 16 polymorphic SSR markers used to test the mutant genotypes. [Genotype information: C) IWP-control; 1)WP-5-1; 2)WP-5-4; 3)WP-6-3; 4)WP-6-4; 5)WP-6-5; 6)WP-15-1; 7)WP-15-5; 8)WP-16-1; 9)WP-16-2; 10)WP-16-3; 11)WP-16-4; 12)WP-16-5; 13)WP-22-1; 14)WP-22-2; 15)WP-22-3; 16)WP-22-5; 17)WP-23-3; 18)WP-23-4; 19)WP-30-1; 20)WP-30-5]
