# Supplementary material for: Assessment of efficacy of mutagenesis of gamma-irradiation in plant height and days to maturity through expression analysis in rice
Source: PLoS One. 2021 Jan 15;16(1):e0245603. doi: 10.1371/journal.pone.0245603 (PMC7810314; doi:10.1371/journal.pone.0245603)
Supplement: S2 Fig — (PDF) [file pone.0245603.s002.pdf]

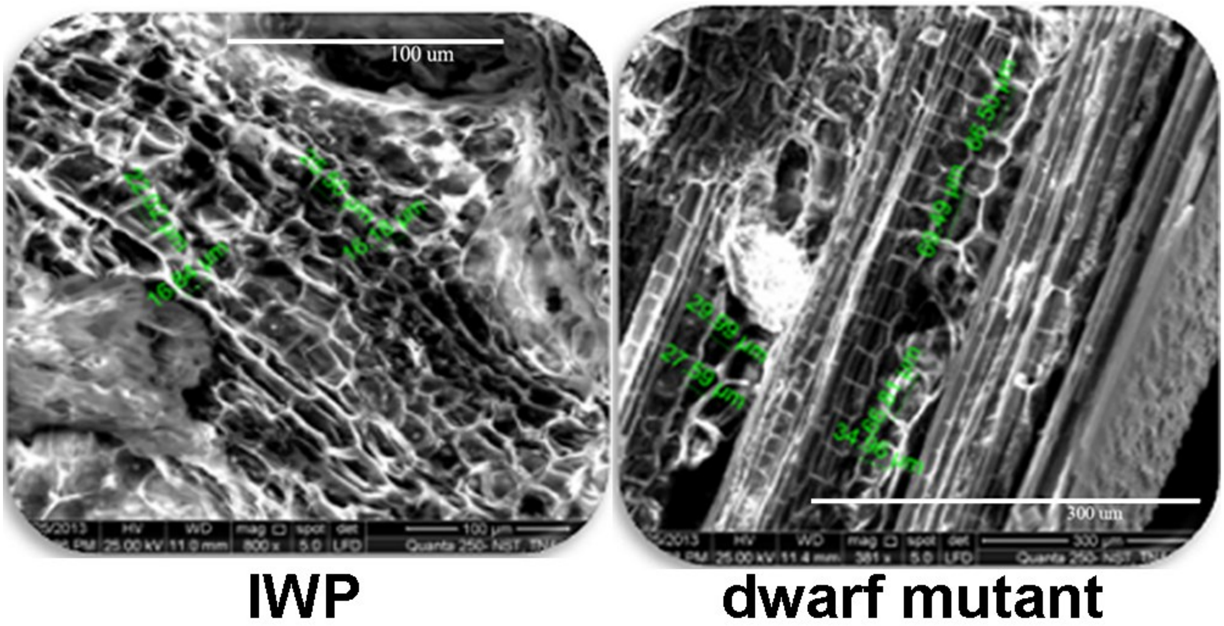

S2 Fig. Scanning electron microscopy of Improved White Ponni and a dwarf mutant

The scanning electron microscopy showing difference in cell size and number of cells per unit area between IWP and a dwarf mutant. Scale bars: IWP-100 µM; dwarf mutant-300 µM.
