# Supplementary material for: Assessment of efficacy of mutagenesis of gamma-irradiation in plant height and days to maturity through expression analysis in rice
Source: PLoS One. 2021 Jan 15;16(1):e0245603. doi: 10.1371/journal.pone.0245603 (PMC7810314; doi:10.1371/journal.pone.0245603)
Supplement: S1 Table — (PDF) [file pone.0245603.s003.pdf]

Supporting information

S1 Table. Categories of rice based on amylose content (%)

**Category**	**Amylose content (%)**
Waxy	1 – 2
Very low amylose	2 – 9
Low	10 – 20
Intermediate	20 – 25
High	25 – 30
