# Supplementary material for: Assessment of efficacy of mutagenesis of gamma-irradiation in plant height and days to maturity through expression analysis in rice
Source: PLoS One. 2021 Jan 15;16(1):e0245603. doi: 10.1371/journal.pone.0245603 (PMC7810314; doi:10.1371/journal.pone.0245603)
Supplement: S2 Table — (PDF) [file pone.0245603.s004.pdf]

S2 Table. List of SSR markers used in this study

S.No.	Name of the primer	Chromosome	Position (cM)	QTL associated	References
1.	RM580	1	68.2	***qPH-1***,*qLFI-1*	[48]
2.	RM246	1	115.2	***qPH1.1***, *qGP-1*	[49]
3.	RM543	1	145.6	***qHd1***	[49]
4.	RM302	1	147.8	*qTGW-1*, ***qDFF1-1***	[50,51]
5.	RM263	2	127.5	***qPh2.2***	[52]
6.	RM250	2	170.1	***qPh2.2***	[52]
7.	RM5430	2	111.5	*wt100-vb2.1*	[53]
8.	RM5699	2	42.1	***qHPH2d***	[54]
9.	RM240	2	158	***qHd2***	[52]
10	RM207	2	191.2	***qHd2***	[52]
11	RM5849	3	18.4	***qHA3-3***	[55]
12	RM7365	3	49.3	*qTGW3-1*	[56]
13	RM545	3	35.3	***qHD-3***	[52]
14	RM7	3	64	*qDTH3.1*	[57]
15	RM3203	3	2.2	***qHd3***	[52]
16	RM252	4	99	***qPh4***	[52]
17	RM567	4	153.6	***qPh4***	[52]

18	RM5709	4	109.9	***qPHT4-3***	[51]
19	RM551	4	0	***qPHT4-1***	[51]
20	RM430	5	78.7	***qPh5***	[52]
21	RM480	5	130.6	***qPh5***	[52]
22	RM541	6	75.5	***qPh6.1***	[52]
23	RM30	6	125.4	***qPh6.1***	[52]
24	RM253	6	37.0	***qHD6.1***	[54]
25	RM7023	6	51.3	*qGT6d*	[58]
26	RM6836	6	54.1	*qGP-6*	[49]
27	RM3183	6	60.8	*qcSDW6, qcTDW6*	[59]
28	RM587	6	10.7	***qPH-6***	[60]
29	RM3431	6	43.4	***qHD1***	[61]
30	RM121	6	43.8	***qHd6***	[49]
31	RM527	6	61.2	*qDF-6, qDM-6,* ***Ph-6.1***	[49]
32	RM2752	7	110.1	*qSLW-7, qDWFL-7-1, qFLA-7-1*	[62]
33	RM5720	7	115.5	*qPE-8*	[63]
34	RM7110	7	56.2	***pHd-4***	[64]
35	RM346	7	78.3	*qPW7.1. qGPP7.1, qYLD7.1, EH7-1*	[65]
36	RM3555	7	105.7	***qPHT7-3***	[51]
37	RM214	7	34.7	***qHD-7***	[52]
38	RM11	7	47	***qHD-7***	[52]
39	RM126	8	57	*qSPP8, qGPP8,* ***qHD8, qPH8***	[60]
40	RM310	8	57	***qPH8c***	[60]
41	RM6208	8	42.9	*qPd-8*	[63]
42	RM6356	8	13.4	*qGT8, qTGW8*	[66]
43	RM38	8	28	***qHd8***	[49]
44	RM25	8	52.2	***qPH8***	[60]
45	RM1019	8	0.5	***qHD-8.1***	[52]
46	RM5432	8	35.7	***qHD-8.1***	[52]
47	RM331	8	60.5	***qHD-8.2***	[52]
48	RM447	8	124.6	***qHD-8.2***	[52]
49	RM3912	9	46.3	***qPh9, qHd9***	[52]

50.	RM278	9	77.5	***qPh9, qHd9***	[52]
51.	RM7173	11	0.4	*qTLNs11, PANNs11*	-
52.	RM332	11	27.9	***qHd11***	[49]
53.	RM167	11	37.5	***qPH11***	[60]

(References in detail, are also quoted in the main text of this manuscript)
