# Supplementary material for: Assessment of efficacy of mutagenesis of gamma-irradiation in plant height and days to maturity through expression analysis in rice
Source: PLoS One. 2021 Jan 15;16(1):e0245603. doi: 10.1371/journal.pone.0245603 (PMC7810314; doi:10.1371/journal.pone.0245603)
Supplement: S3 Table — (PDF) [file pone.0245603.s005.pdf]

S3 Table. Category of IWP and mutants based on amylose content

**S.No.**	**Genotypes**	**Amylose (%) (%)**	**Category**
1.	WP 5-1	33.47	High
2.	WP 5-4	22.32	Intermediate
3.	WP 6-3	16.52	Low
4.	WP 6-4	34.79	High
5.	WP 6-5	36.63	High
6.	WP 15-1	26.17	High
7.	WP 15-5	22.95	Intermediate
8.	WP 16-1	32.67	High
9.	WP 16-2	37.21	High
10.	WP 16-3	35.60	High
11.	WP 16-4	29.10	High
12.	WP 16-5	21.80	Intermediate
13.	WP 22-1	29.96	High
14.	WP 22-2	25.83	High
15.	WP 22-3	34.50	High
16.	WP 22-5	29.33	High
17.	WP 23-3	13.24	Low
18.	WP 23-4	31.23	High
19.	WP 30-1	14.96	Low
20.	WP 30-5	31.46	High
21.	IWP Cont	19.28	Low
