# Supplementary material for: Assessment of efficacy of mutagenesis of gamma-irradiation in plant height and days to maturity through expression analysis in rice
Source: PLoS One. 2021 Jan 15;16(1):e0245603. doi: 10.1371/journal.pone.0245603 (PMC7810314; doi:10.1371/journal.pone.0245603)
Supplement: S4 Table — (PDF) [file pone.0245603.s006.pdf]

S4 Table. Genotypic correlation between traits and their significance

Traits	DFF	PH	NOPT	PL	GPP	TGW	SPY	Mill	HRR	LBC	BBC	LB	LAC	BAC	LER	BER
PH	0.78^{**}															
NOPT	-0.56^{**}	-0.53^{*}														
PL	-0.01^{NS}	0.34^{NS}	0.19^{NS}													
GPP	0.11^{NS}	-0.01^{NS}	-0.19^{NS}	-0.09^{NS}												
TGW	0.12^{NS}	0.49^{*}	-0.21^{NS}	0.38^{NS}	-0.43^{NS}											
SPY	-0.37^{NS}	-0.28^{NS}	-0.14^{NS}	0.02^{NS}	0.18^{NS}	0.10^{NS}										
Mill	-0.35^{NS}	-0.70^{**}	0.43^{NS}	-0.27^{NS}	0.29^{NS}	-0.42^{NS}	0.21^{NS}									
HRR	0.16^{NS}	-0.13^{NS}	-0.22^{NS}	-0.36^{NS}	0.24^{NS}	-0.43^{*}	-0.33^{NS}	0.18^{NS}								
LBC	0.18^{NS}	0.30^{NS}	0.19^{NS}	0.36^{NS}	-0.49^{*}	0.61^{**}	-0.07^{NS}	-0.35^{NS}	-0.49^{*}							
BBC	0.24^{NS}	0.50^{*}	-0.40^{NS}	-0.09^{NS}	-0.14^{NS}	0.65^{**}	0.08^{NS}	-0.43^{NS}	-0.25^{NS}	0.43^{NS}						
LB	0.01^{NS}	-0.09^{NS}	0.52^{*}	0.44^{*}	-0.38^{NS}	0.08^{NS}	-0.15^{NS}	-0.01^{NS}	-0.29^{NS}	0.66^{**}	-0.39^{NS}					
LAC	0.07^{NS}	0.34^{NS}	0.08^{NS}	0.49^{*}	-0.51^{*}	0.86^{**}	0.01^{NS}	-0.19^{NS}	-0.62^{**}	0.73^{**}	0.35^{NS}	0.45^{*}				
BAC	-0.11^{NS}	0.28^{NS}	-0.03^{NS}	0.22^{NS}	-0.09^{NS}	0.48^{*}	-0.21^{NS}	-0.17^{NS}	-0.24^{NS}	0.02^{NS}	0.64^{**}	-0.51^{*}	0.28^{NS}			
LER	-0.11^{NS}	0.14^{NS}	-0.10^{NS}	0.31^{NS}	-0.18^{NS}	0.55^{**}	0.10^{NS}	0.13^{NS}	-0.33^{NS}	-0.09^{NS}	0.01^{NS}	-0.11^{NS}	0.61^{**}	0.39^{NS}		
BER	-0.30^{NS}	0.01^{NS}	0.25^{NS}	0.35^{NS}	-0.02^{NS}	0.14^{NS}	-0.33^{NS}	0.08^{NS}	-0.14^{NS}	-0.28^{NS}	0.10^{NS}	-0.37^{NS}	0.11^{NS}	0.83^{**}	0.50^{*}	
Amylose	0.42^{NS}	0.03^{NS}	-0.56^{**}	-0.58^{**}	0.38^{NS}	-0.29^{NS}	-0.07^{NS}	0.06^{NS}	0.61^{**}	-0.35^{NS}	-0.03^{NS}	-0.33^{NS}	-0.43^{NS}	-0.34^{NS}	-0.23^{NS}	-0.42^{NS}

**-significant at 1% level; *-significant at 5% level; ^{NS}-non-significant.

(DFF- days to fifty per cent flowering; PH-plant height; NOPT-number of productive tillers; PL-panicle length; GPP-grains per panicle; TGW-thousand grain weight; SPY-single plant yield; Mill-milling per cent; HRR- head rice recovery; LBC-length before cooking; BBC-breadth before cooking; LB-L/B ratio; LAC-length after cooking; BAC-breadth after cooking; LER-linear elongation ratio; BER-breadth-wise elongation ratio;
