# Supplementary material for: Assessment of efficacy of mutagenesis of gamma-irradiation in plant height and days to maturity through expression analysis in rice
Source: PLoS One. 2021 Jan 15;16(1):e0245603. doi: 10.1371/journal.pone.0245603 (PMC7810314; doi:10.1371/journal.pone.0245603)
Supplement: S5 Table — (PDF) [file pone.0245603.s007.pdf]

S5 Table. Contribution of traits to the variances of principal components

	PC1	PC2	PC3	PC4	PC5
DFF	0.07	15.50	5.44	4.26	2.00
PH	4.65	16.22	0.87	3.85	0.45
NOPT	0.15	20.90	0.03	3.32	0.69
PL	6.90	0.52	0.66	4.21	19.04
GPP	6.53	1.72	2.62	0.11	11.15
TGW	15.09	2.00	0.15	2.20	0.00
SPY	0.01	0.94	0.25	36.36	3.98
Mill	4.28	7.44	3.38	1.28	2.28
HRR	9.87	2.19	0.05	4.76	0.51
LBC	10.52	0.12	10.27	0.02	0.77
BBC	3.71	9.17	0.10	8.02	5.50
LB	1.92	8.47	11.22	6.13	8.07
LAC	16.69	0.12	0.04	0.39	1.79
BAC	4.26	1.91	20.06	0.89	4.59
LER	4.13	0.00	11.84	0.43	0.65
BER	1.17	0.23	24.64	9.96	0.59
Amylose	7.74	8.44	1.44	0.67	0.17
ASV	2.31	3.29	4.52	6.45	14.77
GC	0.01	0.82	2.43	6.68	23.00
