# Supplementary material for: Assessment of efficacy of mutagenesis of gamma-irradiation in plant height and days to maturity through expression analysis in rice
Source: PLoS One. 2021 Jan 15;16(1):e0245603. doi: 10.1371/journal.pone.0245603 (PMC7810314; doi:10.1371/journal.pone.0245603)
Supplement: S6 Table — (PDF) [file pone.0245603.s008.pdf]

S6 Table. Dissimilarity matrix constructed based on microsatellite marker data

GT	1	2	3	4	5	6	7	8	9	10	11	12	13	14	15	16	17	18	19	20
**2**	0.321																			
**3**	0.321	0.170																		
**4**	0.292	0.142	0.198																	
**5**	0.302	0.142	0.198	0.094																
**6**	0.321	0.123	0.179	0.075	0.047															
**7**	0.330	0.198	0.189	0.132	0.151	0.132														
**8**	0.330	0.142	0.226	0.113	0.113	0.113	0.094													
**9**	0.170	0.283	0.302	0.198	0.245	0.245	0.274	0.236												
**10**	0.330	0.151	0.245	0.160	0.132	0.123	0.123	0.123	0.274											
**11**	0.349	0.151	0.226	0.123	0.094	0.085	0.123	0.085	0.274	0.075										
**12**	0.368	0.170	0.245	0.142	0.132	0.104	0.142	0.104	0.292	0.113	0.038									
**13**	0.358	0.160	0.226	0.151	0.151	0.113	0.189	0.170	0.311	0.179	0.142	0.123								
**14**	0.387	0.151	0.217	0.142	0.113	0.066	0.160	0.142	0.311	0.170	0.132	0.113	0.085							
**15**	0.358	0.151	0.189	0.142	0.151	0.104	0.142	0.142	0.283	0.170	0.132	0.113	0.085	0.038						
**16**	0.425	0.226	0.283	0.217	0.245	0.217	0.283	0.264	0.292	0.255	0.255	0.274	0.236	0.217	0.179					
**17**	0.434	0.226	0.302	0.198	0.217	0.189	0.245	0.208	0.368	0.255	0.179	0.198	0.208	0.198	0.198	0.208				
**18**	0.453	0.255	0.330	0.245	0.217	0.226	0.245	0.189	0.396	0.217	0.179	0.217	0.264	0.255	0.255	0.283	0.179			
**19**	0.415	0.217	0.274	0.189	0.160	0.170	0.189	0.151	0.358	0.160	0.085	0.123	0.208	0.198	0.198	0.302	0.160	0.094		
**20**	0.302	0.377	0.377	0.349	0.358	0.377	0.396	0.368	0.340	0.349	0.368	0.368	0.415	0.425	0.406	0.396	0.406	0.358	0.302	
**21**	0.170	0.396	0.340	0.311	0.358	0.358	0.387	0.406	0.189	0.368	0.387	0.387	0.377	0.406	0.377	0.387	0.406	0.396	0.340	0.113

GT – genotypes; 1. WP-Control, 2. WP 5-1, 3. WP 5-4, 4. WP 6-3, 5. WP 6-4, 6. WP 6-5, 7. WP 15-1, 8. WP 15-5, 9. WP 16-1, 10. WP 16-2, 11. WP 16-3, 12. WP 16-4, 13. WP 16-5, 14. WP 22-1, 15. WP 22-2, 16. WP 22-3, 17. WP 22-5, 18. WP 23-3, 19. WP 23-4, 20. WP 30-1, 21. WP 30-5.
