# Supplementary material for: Assessment of efficacy of mutagenesis of gamma-irradiation in plant height and days to maturity through expression analysis in rice
Source: PLoS One. 2021 Jan 15;16(1):e0245603. doi: 10.1371/journal.pone.0245603 (PMC7810314; doi:10.1371/journal.pone.0245603)
Supplement: S7 Table — (PDF) [file pone.0245603.s009.pdf]

S7 Table. Mean internode and second leaf lengths of IWP, WP-22-2 (untreated) and WP-22-2 (GA3 treated)

	Trait	Mean	Standard Error
IWP (wild-type)	1st internode	6.96	0.29
	2nd leaf	11.20	0.54
	Seedling height	18.16	0.34
WP-22-2 (untreated)	1st internode	5.36	0.09
	2nd leaf	10.26	0.46
	Seedling height	15.62	0.43
WP-22-2 (GA3)	1st internode	6.72	0.41
	2nd leaf	14.90	0.99
	Seedling height	21.62	0.97
