# Supplementary material for: Assessment of efficacy of mutagenesis of gamma-irradiation in plant height and days to maturity through expression analysis in rice
Source: PLoS One. 2021 Jan 15;16(1):e0245603. doi: 10.1371/journal.pone.0245603 (PMC7810314; doi:10.1371/journal.pone.0245603)
Supplement: S8 Table — (PDF) [file pone.0245603.s010.pdf]

S8 Table. Student's t-test for variances of 1st internode and 2nd leaf lengths

	**Test**	**t-stat**	**P value (two-tail)**
Seedling height	IWP vs. WP-22-2 (untreated)	4.61	0.001**
	WP-22-2 (untreated) vs. WP-22-2 (GA3)	-5.62	0.000**
	IWP vs. WP-22-2 (GA3)	-3.35	0.004**
1st internode	IWP vs. WP-22-2 (untreated)	5.21	0.003**
	WP-22-2 (untreated) vs. WP-22-2 (GA3)	-3.23	0.005**
	IWP vs. WP-22-2 (GA3)	0.48	0.638^{ns}
2nd leaf	IWP vs. WP-22-2 (untreated)	1.33	0.219^{ns}
	WP-22-2 (untreated) vs. WP-22-2 (GA3)	-4.26	0.000**
	IWP vs. WP-22-2 (GA3)	-3.28	0.004**
