# Supplementary material for: Assessment of efficacy of mutagenesis of gamma-irradiation in plant height and days to maturity through expression analysis in rice
Source: PLoS One. 2021 Jan 15;16(1):e0245603. doi: 10.1371/journal.pone.0245603 (PMC7810314; doi:10.1371/journal.pone.0245603)
Supplement: S9 Table — (PDF) [file pone.0245603.s011.pdf]

S9 Table. The raw C_T values and the calculated $\Delta\Delta C_T$ values observed for Improved White Ponni

Detector	Sample	Raw C_T			Mean	SD	Endogenous C_T(Control)	ΔC_T	$\Delta\Delta C_T$
		R1	R2	R3					
Actin	0h	18.69	18.75	18.87	18.77	0.09	18.77	0.00	0.00
	6h	19.71	18.9	18.68	19.11	0.54	19.11	0.00	0.00
	12h	17.52	17.56	17.85	17.64	0.18	17.64	0.00	0.00
	24h	17.99	17.54	17.74	17.76	0.22	17.76	0.00	0.00
SLR1	0h	20.28	20.32	18.97	19.86	0.77	18.77	1.09	0.00
	6h	20.46	19.36	19.96	19.93	0.55	19.11	0.82	-0.27
	12h	19.65	19.55	19.70	19.63	0.08	17.64	1.99	0.90
	24h	19.48	19.38	19.74	19.54	0.19	17.76	1.78	0.69
GA	0h	25.62	24.92	25.51	25.35	0.37	18.77	6.58	0.00
	6h	25.67	28.25	25.47	26.46	1.55	19.11	7.35	0.78
	12h	25.78	25.27	25.24	25.43	0.30	17.64	7.79	1.21
	24h	25.32	25.26	25.77	25.45	0.28	17.76	7.69	1.12
OsKOL4	0h	18.92	16.79	18.87	18.19	1.22	18.77	-0.58	0.00
	6h	19.85	18.63	19.21	19.23	0.61	19.11	0.12	0.70
	12h	17.69	18.62	18.38	18.22	0.48	17.64	0.58	1.16
	24h	18.26	19.12	19.87	19.08	0.80	17.76	1.33	1.90
KO2	0h	23.87	23.40	23.56	23.61	0.24	18.77	4.84	0.00
	6h	22.70	19.95	22.72	21.79	1.60	19.11	2.68	-2.16
	12h	21.64	21.77	21.90	21.77	0.13	17.64	4.13	-0.71
	24h	22.95	23.55	23.15	23.22	0.31	17.76	5.46	0.62
MAX2	0h	27.79	28.95	27.79	28.18	0.67	18.77	9.41	0.00
	6h	27.27	28.03	27.88	27.73	0.40	19.11	8.62	-0.79
	12h	26.98	26.06	27.02	26.69	0.54	17.64	9.04	-0.36
	24h	26.89	25.98	26.84	26.57	0.51	17.76	8.81	-0.59
OsBRD2	0h	24.81	24.25	24.96	24.67	0.37	18.77	5.90	0.00
	6h	24.70	25.60	24.50	24.93	0.58	19.11	5.82	-0.08
	12h	22.36	22.64	22.65	22.55	0.16	17.64	4.90	-1.001
	24h	22.38	22.86	22.65	22.63	0.24	17.76	4.88	-1.02
