# Supplementary material for: Assessment of efficacy of mutagenesis of gamma-irradiation in plant height and days to maturity through expression analysis in rice
Source: PLoS One. 2021 Jan 15;16(1):e0245603. doi: 10.1371/journal.pone.0245603 (PMC7810314; doi:10.1371/journal.pone.0245603)
Supplement: S10 Table — (PDF) [file pone.0245603.s012.pdf]

S10 Table. The raw C_T values and the calculated $\Delta\Delta C_T$ values observed for WP-22-2

Detector	Sample	Raw C_T			Mean	SD	Endogenous C_T(Control)	ΔC_T	$\Delta\Delta C_T$
		R1	R2	R3					
Actin	0h	17.91	16.96	17.59	17.49	0.48	17.49	0.00	0.00
	6h GA	17.95	17.92	17.93	17.93	0.01	17.93	0.00	0.00
	12h GA	18.09	17.92	17.84	17.95	0.13	17.95	0.00	0.00
	24h GA	17.80	17.94	17.86	17.87	0.07	17.87	0.00	0.00
SLR1	0h	19.74	18.52	19.38	19.21	0.63	17.49	1.72	0.00
	6h GA	19.95	20.47	20.27	20.23	0.26	17.93	2.30	0.57
	12h GA	19.44	19.05	19.43	19.31	0.22	17.95	1.36	-0.36
	24h GA	19.36	19.39	19.52	19.42	0.08	17.87	1.55	-0.17
GA	0h	24.05	24.08	23.51	23.88	0.32	17.49	6.39	0.00
	6h GA	25.99	27.03	25.96	26.33	0.61	17.93	8.40	2.00
	12h GA	25.55	24.11	25.85	25.17	0.93	17.95	7.22	0.83
	24h GA	25.55	25.58	25.42	25.52	0.08	17.87	7.65	1.26
OsKOL4	0h	18.19	18.50	19.26	18.65	0.55	17.49	1.16	0.00
	6h GA	19.50	19.83	19.55	19.62	0.18	17.93	1.69	0.53
	12h GA	19.02	19.45	19.91	19.46	0.45	17.95	1.51	0.35
	24h GA	19.75	19.60	19.71	19.69	0.08	17.87	1.82	0.66
KO2	0h	22.31	22.55	22.27	22.38	0.15	17.49	4.89	0.00
	6h GA	27.69	27.64	28.67	23.22	0.16	17.93	4.89	0.40
	12h GA	27.45	27.85	27.33	22.54	0.17	17.95	4.89	-0.30
	24h GA	26.73	26.50	27.03	23.48	0.08	17.87	4.89	0.73
MAX2	0h	27.26	27.71	27.24	27.40	0.26	17.49	9.92	0.00
	6h GA	19.50	19.83	19.55	28.00	0.58	17.93	9.92	0.15
	12h GA	19.02	19.45	19.91	27.54	0.28	17.95	9.92	-0.32
	24h GA	19.75	19.60	19.71	26.76	0.26	17.87	9.92	-1.03
OsBRD2	0h	22.83	23.17	23.27	23.09	0.23	17.49	5.60	0.00
	6h GA	24.39	24.40	24.30	24.36	0.06	17.93	5.60	0.83
	12h GA	23.64	23.90	23.40	23.65	0.25	17.95	5.60	0.09
	24h GA	24.37	24.50	24.65	24.51	0.14	17.87	5.60	1.03
